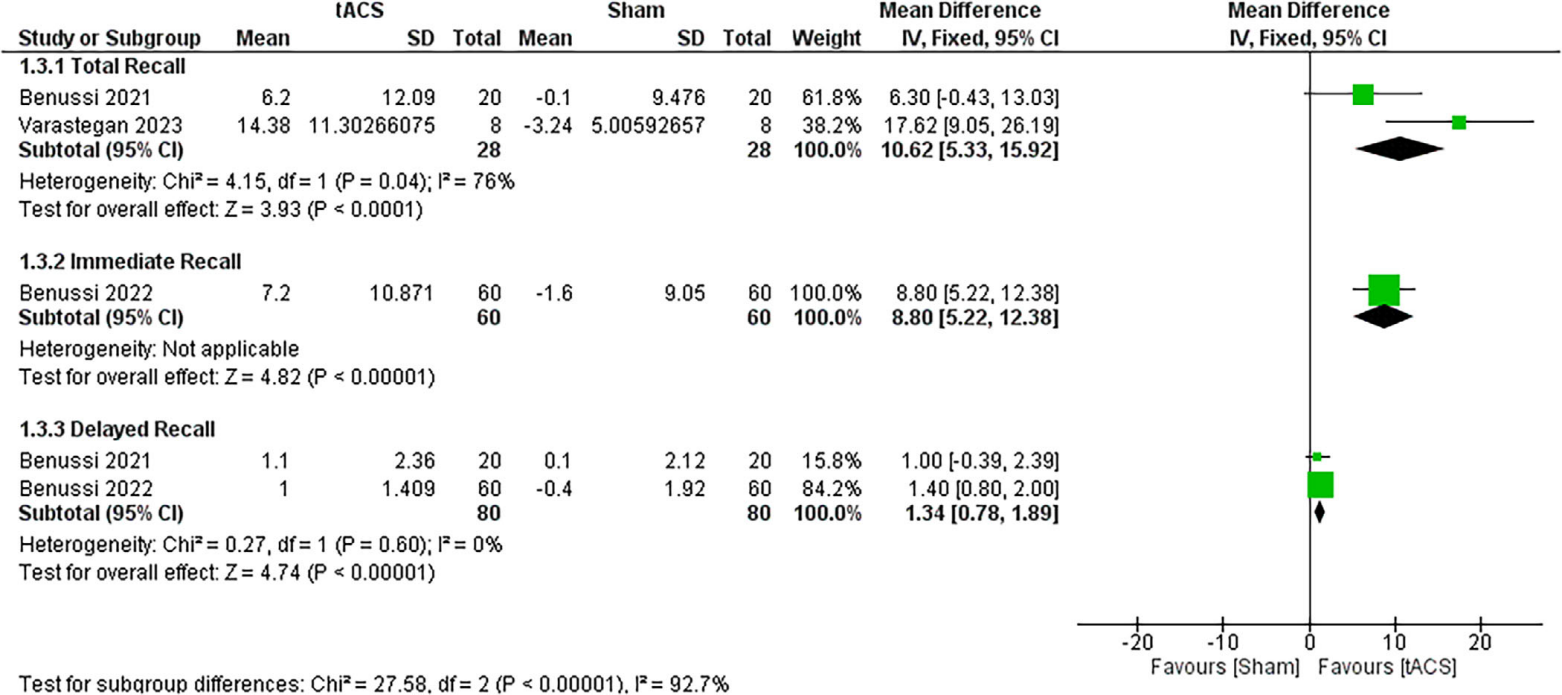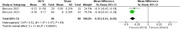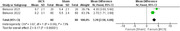# Efficacy of Transcranial Alternating Current Stimulation (tACS) in Cognition and Memory in Patients with Mild Cognitive Impairement or Alzheimer’s Disease. A Systematic Review and Meta‐analysis of Randomized Controlled Trials

**DOI:** 10.1002/alz.087903

**Published:** 2025-01-09

**Authors:** Nada Ibrahim Hendi, Mohamed El‐Samahy, Yosra Hussein AboEl‐Azm, Amina Arar, Shrouk I Ramadan, Esraa M Zedan, Noha Samy Yasen

**Affiliations:** ^1^ Faculty of Medicine, Ain Shams University, Cairo Egypt; ^2^ Faculty of Medicine, Zagazig University, Sharkeya Egypt; ^3^ Faculty of Medicine, Algiers University, Algiers Algeria; ^4^ Faculty of Medicine, Al‐Azhar University, Cairo Egypt; ^5^ Applied Medical Sciences, Misr University for Science and Technology, Cairo Egypt

## Abstract

**Background:**

Gamma desynchronization is an early pathophysiological event in Alzheimer’s disease with a disturbance in oscillation in the gamma frequency range 30‐80 Hz. This disruption was found to be directly related to the disease progression and severity. Thus, the use of transcranial alternating current stimulation (tACS) possessed greater interest. tACS can induce synchronization of neural networks leading to improvement in behavior and cognition. However, there’s still controversy regarding the beneficial effect of tACS in improving cognition and memory in Alzheimer’s disease patients. This systematic review and meta‐analysis aims to pool and synthesize evidence from randomized controlled trials to assess the efficacy of tACS on memory and cognition in patients with mild cognitive impairment or Alzheimer’s disease.

**Method:**

We searched PubMed, Scopus, Web of Science up to Dec 17, 2023. We included a total of seven randomized controlled trials comprising 246 patients with Alzheimer’s disease or mild cognitive impairment. Our primary outcomes were Rey auditory verbal learning scale (RAVL), short latency afferent inhibition (SAI), and face‐name association task (FNAT).

**Result:**

There was a significant difference between tACS and sham groups in favor of tACS in RAVL immediate recall (MD = 8.80, 95% CI = [5.22, 12.38], P < 0.00001), delayed recall (MD = 1.34, 95% CI = [0.78, 1.89], P < 0.00001), and total recall (MD = 10.62, 95% CI = [5.33, 15.92], P < 0.00001). Short latency afferent inhibition was significantly reduced in the tACS group compared to sham group (MD = ‐0.35, 95% CI = [‐0.41, ‐0.29], P < 0.00001). There was also a significant difference in FNAT in favor of tACS group (MD = 3.29, 95% CI = [2.50, 4.08], P < 0.00001)

**Conclusion:**

Preliminary evidence suggests the potential efficacy of tACS for improving cognition in Alzheimer’s disease patients. However, due to the limited number of studies and the small sample size, there’s still insufficient evidence to conclude a decision regarding this matter. More standardized, large clinical trials are needed to establish better evidence.